# Supplementary material for: Reversible morphological changes in a juvenile marine fish after exposure to predatory alarm cues
Source: R Soc Open Sci. 2020 May 13;7(5):191945. doi: 10.1098/rsos.191945 (PMC7277257; doi:10.1098/rsos.191945)

Fig. S1. Position of the 10 landmarks used to analyze the shape of gilthead seabream within the paper by Díaz-Gil et al: Reversible morphological changes in a juvenile marine fish after exposure to predatory alarm cues. Royal Society Open Science

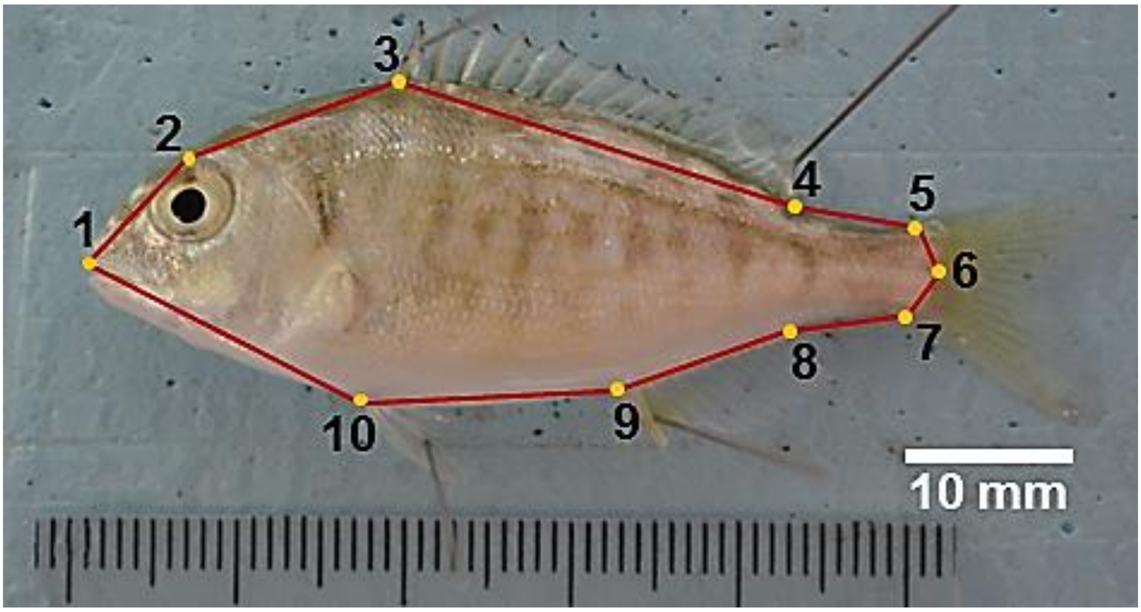

Supplement: Landmarks used for the shape analysis [file RSOS191945supp1.pdf]
