# Supplementary material for: Reversible morphological changes in a juvenile marine fish after exposure to predatory alarm cues
Source: R Soc Open Sci. 2020 May 13;7(5):191945. doi: 10.1098/rsos.191945 (PMC7277257; doi:10.1098/rsos.191945)
Supplement: Experimental design, analyses and raw data [file RSOS191945supp2.doc]

**Supplementary material**

**Index**

1. **Experimental design**

(a) Fish obtention, acclimation and feeding

(b) Preparation of chemical cues

(c) Experiment

1. **Analyses**

(a) General growth and condition descriptors

(b) Geometric morphometrics

1. ***Raw data***
2. ***References***
3. **Experimental design**
4. Fish obtention, acclimation and feeding

The 500 juvenile fish were obtained from a wild-type stock reared at a local aquaculture station, Aquicultura Balear (S.A.U., CULMAREX Group, Balearic Islands, Spain). Fish were transported to the laboratory in <1h in temperature and oxygen-controlled tanks. Acclimatisation to the laboratory conditions implied an only 1°C change and no salinity change with respect to the aquaculture station. Experimental conditions were: T = 20 °C ± 0.5 °C, salinity = 36.8 ± 0.01, natural photoperiod. The rest of the individuals were kept in a similar tank for other procedures. Fish were fed ad libitum with commercial food pellets for sea bream juveniles (1.5 mm, Perla MP, Skretting Spain S.A.) during all the experiments at the recommended doses for this life stage.

1. Preparation of chemical cues

The chemical cues of conspecific exposed to predators was obtained using 20 individuals (mean total length 10 ± 2 cm) of their natural predator [1], the wild black scorpionfish (*Scorpaena porcus,*). Wild *S. porcus* specimens were captured using an experimental beam trawl [2] and maintained in captivity in an oxygen-saturated open flow aquarium (80 L) and fed daily with five live *S. aurata*. The water samples used to extract the chemical cues of predators and prey were filtered through 53 microns mesh to remove large particles. The total weight of the skins used to fraction the samples with the chemical cues amounted to approximately 10 g (20 fish). The predatory chemical cues were prepared into individual 200 ml zipped plastic bags, kept frozen until the experiment. To intensify the scent of conspecifics, as recommended by the literature, the skin of the 20 euthanised *S. aurata* was homogenised in 3 L of seawater, and fractioned in 50 ml doses that were added to the predatory cues.

1. Experiment

During the first month of the experiment, the water flow of each tank was turned off at 10:00 AM daily. One ice cube of 200 mL (control or predator water, as corresponding) was incorporated into each tank from monday to friday; the water flow remained off during 4 hours so that the chemical cue spreaded in the tank. The water flow was then turned on again to ~4 L h^-1^. This represented 20 doses per tank during the first month. During the second month, the fish were kept under equal conditions but without adding any chemical cue.

1. **Analyses**
2. General growth and condition descriptors

Prior to the analyses, a few size outliers were removed to select proper size groups. All analyses were conducted separately for each sampling time. The comparison between treatment and sampling times was conducted through Linear mixed-effect model (LMM) with Satterthwaite approximations to degrees of freedom using *lmer* function from *lme4* package [3] in R software [4], to include the random effects of the different tanks. In all the cases the assumptions of the model were checked and data natural log-transformed if necessary and, prior to the analyses, a few outliers were removed to select proper size groups within each sampling date [5].

1. Geometric morphometrics

Standardised photographs of every sampled fish for geometric morphometric analyses were taken with a Nikon D3100 camera, from the exact same fixed height, focal distance and including a ruler for proper calibration in all the cases. The body shape of each individual was analysed using the landmark-based method [6]. A total of 10 homologous landmarks were selected based on a previous study with this species [7] (see Fig.S1). The *xy* coordinates of these landmarks of each individual were acquired from a lateral (left side) photograph of the fish using the tpsDig2 software [8]. The raw coordinates were superimposed using General Procrustes Alignment (GPA) as implemented in the function *gpagen* from the *geomorph* library in R [9]. Afterwards a multivariate linear regression via Procrustes distances ANOVA [10] was performed on the superimposed coordinates used as shape descriptors using a residual randomization permutation procedure to evaluate the observed sum-of-squared of Procrustes distances [11]. We specifically used *procD.allometry* function which accounts for the confounding effect of the fish size (included as the natural logarithm of the centroid size) on the body shape (allometry) [9,12]. This allowed us to visualise and remove the effect of the different sizes on the body shape, a widespread property of organisms [12], and focus on the effect of the treatment. The final comparison of changes in allometry-free body shape is detailed in the paper.

1. **Raw data**

Raw data are supplied as a separate excel file. The file contains two spreadsheets. One with data on length and weight, and another one with the scale-corrected landmarks to enable the reproducibility of the shape analysis.

Variables for length and weight contain an identifier for sampling period (Sampling, 1, 2 or 3), Treatment (C=control, T=treatment), fish identifier (Fish_ID, a number), length (TL_mm, total length in mm), and weight (W_g, in grams).

The landmarks spreadsheet contain the same variables for sampling period, treatment and fish identifier, plus the x and y coordinates (arbitrary units) of the 10 landmarks.

1. **References**

1. Deudero S, Morey G, Frau A, Moranta J, Moreno I. 2008 Temporal trends of littoral fishes at deep *Posidonia oceanica* seagrass meadows in a temperate coastal zone. *J. Mar. Syst.* **70**, 182–195. (doi:10.1016/j.jmarsys.2007.05.001)

2. Catalán IA, Dunand A, Álvarez I, Alós J, Colinas N, Nash RDM. 2014 An evaluation of sampling methodology for assessing settlement of temperate fish in seagrass meadows. *Mediterr. Mar. Sci.* **15**, 338–349.

3. Bates D, Mächler M, Bolker B, Walker S. 2015 Fitting Linear Mixed-Effects Models using lme4. *J. Stat. Softw.* **67**, 48. (doi:10.18637/jss.v067.i01)

4. R Development Core Team, Team RDC. 2011 *R: A language and environment for statistical computing.* Vienna, Austria. (doi:10.1007/978-3-540-74686-7)

5. Zuur AF, Ieno EN, Elphick CS. 2010 A protocol for data exploration to avoid common statistical problems. *Methods Ecol. Evol.* **1**, 3–14. (doi:10.1111/j.2041-210X.2009.00001.x)

6. Rohlf FJ, Marcus LF. 1993 A revolution in morphometrics. *Trends Ecol. Evol.* **8**, 129–132.

7. Verhaegen Y, Adriaens D, Wolf T De, Dhert P, Sorgeloos P. 2007 Deformities in larval gilthead sea bream (*Sparus aurata*): A qualitative and quantitative analysis using geometric morphometrics. *Aquaculture* **268**, 156–168. (doi:10.1016/j.aquaculture.2007.04.037)

8. Rohlf FJ. 2004 tpsDig, digitize landmarks and outlines.

9. Adams DC, Otárola-Castillo E. 2013 Geomorph: an R package for the collection and analysis of geometric morphometric shape data. *Methods Ecol. Evol.* **4**, 393–399. (doi:10.1111/2041-210X.12035)

10. Goodall C. 1991 Procrustes methods in the statistical analysis of shape. *J. R. Stat. Soc.* **53**, 285–339. (doi:10.2307/2345744)

11. Collyer ML, Sekora DJ, Adams DC. 2015 A method for analysis of phenotypic change for phenotypes described by high-dimensional data. *Hered.* **115**, 357–365. (doi:10.1038/hdy.2014.75)

12. Adams DC, Rohlf FJ, Slice DE. 2013 A field comes of age: Geometric morphometrics in the 21st century. *Hystrix* **24**, 7–14. (doi:10.4404/hystrix-24.1-6283)
